# Influence of type 2 diabetes and obesity on adipose mesenchymal stem/stromal cell immunoregulation

**DOI:** 10.1007/s00441-023-03801-6

**Published:** 2023-07-18

**Authors:** Marwa Mahmoud, Mazen Abdel-Rasheed

**Affiliations:** 1 Stem Cell Research Group, Medical Research Centre of Excellence, National Research Centre, 33 El Buhouth St, Ad Doqi, Dokki, 12622 Cairo Governorate Egypt; 2 Department of Medical Molecular Genetics, Human Genetics and Genome Research Institute, National Research Centre, Cairo, Egypt; 3 Department of Reproductive Health Research, National Research Centre, Cairo, Egypt

**Keywords:** Type 2 diabetes, Obesity, Adipose, Mesenchymal, Immunoregulation, Preconditioning

## Abstract

Type 2 diabetes (T2D), associated with obesity, represents a state of metabolic inflammation and oxidative stress leading to insulin resistance and progressive insulin deficiency. Adipose-derived stem cells (ASCs) are adult mesenchymal stem/stromal cells identified within the stromal vascular fraction of adipose tissue. These cells can regulate the immune system and possess anti-inflammatory properties. ASCs are a potential therapeutic modality for inflammatory diseases including T2D. Patient-derived (autologous) rather than allogeneic ASCs may be a relatively safer approach in clinical perspectives, to avoid occasional anti-donor immune responses. However, patient characteristics such as body mass index (BMI), inflammatory status, and disease duration and severity may limit the therapeutic utility of ASCs. The current review presents human ASC (hASC) immunoregulatory mechanisms with special emphasis on those related to T lymphocytes, hASC implications in T2D treatment, and the impact of T2D and obesity on hASC immunoregulatory potential. hASCs can modulate the proliferation, activation, and functions of diverse innate and adaptive immune cells via direct cell-to-cell contact and secretion of paracrine mediators and extracellular vesicles. Preclinical studies recommend the therapeutic potential of hASCs to improve inflammation and metabolic indices in a high-fat diet (HFD)-induced T2D disease model. Discordant data have been reported to unravel intact or detrimentally affected immunomodulatory functions of ASCs, isolated from patients with obesity and/or T2D patients, in vitro and in vivo. Numerous preconditioning strategies have been introduced to potentiate hASC immunomodulation; they are also discussed here as possible options to potentiate the immunoregulatory functions of hASCs isolated from patients with obesity and T2D.

## Type 2 diabetes pathogenesis: role of inflammation and oxidative stress

Type 2 diabetes (T2D) is the predominant form of diabetes mellitus, accounting for nearly 85–90% of all diabetics (Zhou et al. 2016; Khan et al. 2019). T2D is a multifactorial hyperglycemic state in which imbalanced metabolic and inflammatory pathways are integrated to initiate insulin resistance (IR), compensated by hypersecretion of insulin, leading to exhaustion/deterioration of β cells (Coope et al. 2016). Risk factors of T2D, in addition to genetic predisposition, include sedentary lifestyle factors such as excessive dietary intake, physical inactivity, smoking and/or alcohol consumption, environmental factors such as pollution, and psychosocial stress factors. All such factors can modulate the immune system and activate inflammation (Tsalamandris et al. 2019; Galicia-Garcia et al. 2020).

Ample data indicate a strong pathogenic link between obesity, IR, and T2D (Nikolajczyk et al. 2011; Tsalamandris et al. 2019; Roden and Shulman 2019). Obesity induces a chronic state of low-level systemic inflammation, in which adipose tissue plays a key role to mediate (Tsalamandris et al. 2019; Roden and Shulman 2019). In response to elevated lipid and other metabolic fuels, adipose tissue cellular components, mainly hypertrophic adipocytes in concert with accumulated innate (macrophages) (McLaughlin et al. 2017) and adaptive (B and T lymphocytes) immune cells (Xia et al. 2017; McLaughlin et al. 2017) overproduce inflammatory mediators as tumor necrosis factor alpha (TNF-ɑ), interleukin- 6 (IL-6), monocyte chemoattractant protein (MCP), and interleukin-1 beta (IL-1β). These pro-inflammatory factors, among others, are released in circulation to interfere with insulin signaling in insulin-responsive tissues, such as liver and skeletal muscles, and to deteriorate islet β-cell functions leading to defective insulin secretion (Wellen and Hotamisligil 2005; Coope et al. 2016; Galicia-Garcia et al. 2020). Inflammatory mediators activate diverse inflammation pathways such as the nuclear factor kappa-light-chain-enhancer of activated B cells (NF-κB) and c-Jun N-terminal kinase (JNK) signaling pathways which can interfere with insulin tyrosine phosphorylation leading to the development of IR and ultimately T2D (Coope et al. 2016).

Hyperglycemia is closely linked with mitochondrial dysfunction and elevated generation of reactive oxygen species (ROS) in many tissues, which may enhance oxidative stress. Oxidative stress, in turn, aggravates inflammation, IR, and β-cell dysfunction (Rehman and Akash 2017; Ma et al. 2018). Hyperglycemia, oxidative stress, and inflammation in T2D contribute to vascular and non-vascular complications, causing socio-economic burdens (Vikram et al. 2014; Galicia-Garcia et al. 2020; Charlton et al. 2020). Accordingly, approaches to combat inflammation, immune dysregulation, and oxidative stress are potentially recommended to improve glucose metabolism in patients with T2D (Coope et al. 2016; Kitada et al. 2019).

## Adipose tissue: a reservoir for mesenchymal stem/ stromal cells

Adipose tissue (AT) is found throughout the adult human body to serve as an energy reservoir and an endocrine organ and is composed of different cell components, including mesenchymal stromal/stem cells (MSCs) (Bunnell 2021). AT, as an MSCs source, offers numerous advantages over bone marrow (BM), the gold standard source, like easy accessibility, less invasiveness, minimal risk for donor, and higher stem cell yield (Hass et al. 2011). From 1 g of AT, 5 × 10^3^ MSC can be isolated, which is 500 times more cells than from an equivalent amount of BM (Fraser et al. 2006). Adipose-derived MSCs (ASCs), also termed adipose-derived stromal/stem cells, are commonly isolated from the stromal vascular fraction (SVF) of subcutaneous white AT obtained from the abdomen, thigh, or hips by enzymatic digestion with collagenase followed by centrifugation and washing (Zuk et al. 2002).

## ASC characteristics

The ASCs are expandable fibroblast-like cell, typically characterized according to the International Society of Cell & Gene Therapy (ISCT) (Dominici et al. 2006), and the International Federation for Adipose Therapeutics and Science (IFATS) (Bourin et al. 2013), by three criteria (1) the potential to adhere to a plastic surface, (2) they express a panel of surface protein markers; they are positive for CD90, CD105, CD73, CD44, CD29, and CD13, while they are negative for CD14, CD19, CD31, CD34, CD45, and HLA-DR, (3) they can differentiate into osteogenic, adipogenic and chondrogenic lineages in the presence of the respective proper stimuli.

In addition to their ease of isolation, expandability in vitro to give a suitable cell number for clinical use, and multi-differentiation capability (Patrikoski et al. 2013), ASCs are widely reported not to induce the immune response of allogeneic lymphocytes (McIntosh et al. 2006; Cui et al. 2007; Menard et al. 2013). Such property was attributed to the low expression of major histocompatibility Class II molecule (MHC II/HLA-DR) and co-stimulatory molecules, CD40, CD80, and CD86 (Menard et al. 2013; Buyl et al. 2020). On the contrary, some studies illustrated that ASCs had the potential to activate the proliferation of resting allogeneic CD4 T cells under circumstances of low inflammation (Crop et al. 2010; Frazier et al. 2014), or the production of alloreactive- memory CD8 T cells (Chang et al. 2020), so they are not intrinsically immunoprivileged (Ankrum et al. 2014).

## hASC immunoregulatory and anti-inflammatory properties

Human ASCs represent a promising treatment for a wide range of inflammatory and autoimmune diseases (Bateman et al. 2018; Patrikoski et al. 2019) as they harbor multifaceted anti-inflammatory and immunomodulatory properties (Melief et al. 2013a; Ceccarelli et al. 2020). In particular, the immunoregulatory effects of hASCs on effector T cells have been widely studied (Najar et al. 2010a, b; Kronsteiner et al. 2011; Rubtsov et al. 2017; Zhou et al. 2018; Fiori et al. 2021). hASCs can modulate allogeneic- or mitogenic-induced activation (Kuca-Warnawin et al. 2020), proliferation (Fiori et al. 2021), and cytokine secretion (Zhou et al. 2018; Fiori et al. 2021) of T lymphocytes. In addition, hASCs have been reported to affect the proliferation, differentiation, and immune functions of B cells (Bochev et al. 2008; Franquesa et al. 2012), inhibit differentiation and maturation of dendritic cells (DCs) (Ivanova-Todorova et al. 2009; Melief et al. 2013a), suppress natural killer cells (NK) cytotoxicity (DelaRosa et al. 2012; Menard et al. 2013; Ribeiro et al. 2013), and stimulate macrophages polarization to anti-inflammatory macrophages with immunoregulatory functions (M2 macrophages) at the expense of the proinflammatory phenotype (M1 macrophages) (Wang et al. 2019; Zhu et al. 2021).

## Mechanisms underlying hASC immunosuppressive effects

**Fig. 1:**
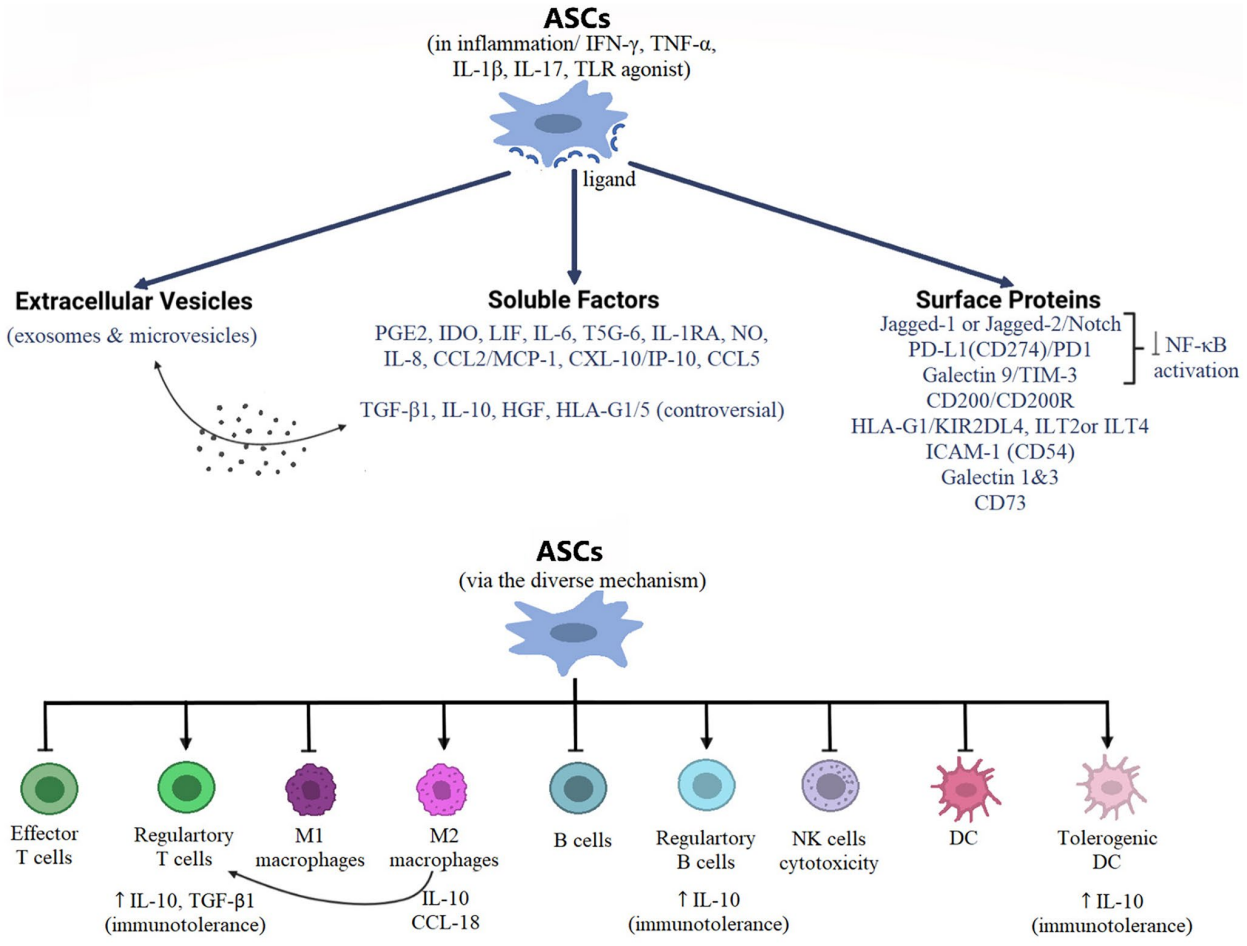
ASC immunoregulatory mechanisms and effects. ASCs are activated in inflammation to express several immunomodulators promoting the regulatory phenotypes, at the expense of the inflammatory ones, of different immune cells, leading finally to immunotolerance. Abbreviations: ASCs, adipose mesenchymal stem/stromal cells; IFN-ɤ, interferon-gamma; TNF-ɑ, tumor necrosis factor alpha; IL, interleukin; TLR, Toll-like receptor; PGE2, prostaglandin E2; IDO, indoleamine 2, 3 dioxygenase 1; LIF, leukemia inhibitory factor; TSG-6, tumor necrosis factor stimulated-gene 6; IL-1RA, interleukin 1 receptor antagonist; NO, nitric oxide; MCP, monocyte chemoattractant protein; IP-10, interferon gamma-inducible protein 10, transforming growth factor beta 1; HGF, hepatocyte growth factor; HLA-G5, human leukocyte antigen-G1/5; PD-L1, programmed death ligand 1; TIM-3, T-cell immunoglobulin and mucin-containing protein 3; CD, cluster of differentiation; ILT 2 or 4, human inhibitory receptors Ig-like transcript 2 or 4; KIR2DL4, killer cell Ig-like receptor, two Ig domains and long cytoplasmic tail 4; ICAM-1, intracellular adhesion molecule 1 l; NF-κB, NF-K nuclear factor kappa-light-chain-enhancer of activated B cells; DC, dendritic cells; NK, natural killer

The mechanisms by which hASCs modulate diverse innate and adaptive immune arms rely mainly on direct cell-to-cell interaction and/or release of soluble factors and/or microvesicles with immunoregulatory potentials (Fig. 1) (Ceccarelli et al. 2020; Hao et al. 2021). The involvement of soluble mediators in MSC- based therapy has been proved by the ability of MSCs to prolong graft survival, despite surviving not longer than 24 h in vivo (Eggenhofer et al. 2012).

Regarding hASCs, numerous soluble factors have been shown to mediate significantly their immunoregulatory effects (Yoo et al. 2009; DelaRosa et al. 2009; Najar et al. 2010a, b; Yañez et al. 2010; Melief et al. 2013a; Rubtsov et al. 2017; Fiori et al. 2021; Kuca-Warnawin et al. 2021b). However, crosstalk between hASCs and PBMCs/immune cells is necessary to release those suppressive factors by hASCs (Yoo et al. 2009; DelaRosa et al. 2009; Najar et al. 2010b). Active communication between ASCs and T cells, where vesicular or nanotube-mediated transfer of components of activated T cells to hASCs, is responsible for shifting the latter population to acquire an immune-regulatory/suppressive phenotype (Matula et al. 2016).

One of the most widely accepted candidates to mediate hASCs immunomodulation is the lipid mediator prostaglandin E2 (PGE2) (Cui et al. 2007; DelaRosa et al. 2009; Najar et al. 2010a; Yañez et al. 2010; Matula et al. 2016; Kuca-Warnawin et al. 2021b). PGE2 is produced from the conversion of arachidonic acid via the inducible expression of the cyclooxygenase (COX-2) enzyme in hASCs. The addition of indomethacin, a PGE2 inhibitor, in the co-culture settings of hASCs and DCs or T cells, abrogated the inhibitory effect of hASCs on the maturation of plasmacytoid, but not myeloid, DCs. PGE2 blockade by indomethacin maintained phytohaemagglutinin (PHA)-activated T lymphocyte proliferation but did not restore the secretion pattern of the pro-inflammatory cytokines; IL-6, TNF-α, interferon-gamma (IFN-γ) and IL-12 by the PHA- T cells. However, PGE2 inhibition resulted in an increased expression of transcription factors and cytokines genes involved in the Th1/Th2 differentiation pathway. The latter results were detected in the PHA-T cells co-cultured with ASCs, but not with BM-MSCs (Yañez et al. 2010). Neutralization assays also provided evidence for the crucial role of PGE2 to mediate the immunoregulatory effects of hASCs on monocyte derivatives resulting in the polarization of macrophages and DCs toward an anti-inflammatory and phagocytic phenotype (Ortiz-Virumbrales et al. 2020). PGE2 was proven to induce macrophages to produce anti-inflammatory IL-10, which in turn, suppressed the proliferation of NK cells (Eleuteri and Fierabracci 2019) and Th cells (Yang et al. 2018). In addition, PGE2 is majorly involved in the regulation of Th17 differentiation (Boniface et al. 2009; Qu et al. 2012).

hASCs and WJ-MSCs have been shown to possess more potent immunosuppressive effects than BM-MSCs via expression and secretion of the immunomodulator; leukemia inhibitory factor (LIF) and suppression of this factor abrogated the antiproliferative effect of hASCs on activated T lymphocytes (Najar et al. 2010b). LIF is a cytokine that mediates differentiation and growth of different cell types (hematopoietic cells, neurons) but also acts as a chemoattractant for neutrophils, eosinophils, and monocytes. LIF can induce IL-8 secretion (Musso et al. 1995), and its production is induced by IL-6 (West 2019), which is considerably secreted by ASCs (Melief et al. 2013a). Additionally, LIF has been shown to interfere with Th17 differentiation (Cao et al. 2011) and induce Tregs expansion (Nasef et al. 2008; Najar et al. 2010b).

The higher expression of IL-6 and TGF-β1 by hASCs contributed to their superior potential over BM-MSCs, to inhibit the differentiation of monocytes into DCs and suppress the proliferation of activated PBMCs (Melief et al. 2013a). It also could skew the monocytes toward an anti-inflammatory IL-10-producing phenotype (Melief et al. 2013b). As well, the IL-6 may increase the secretion of other immunosuppressive factors, such as inducible nitric oxide synthase (iNOs) and PGE2, by MSCs in an autocrine-dependent mechanism (Bouffi et al. 2010). Controversial data have been reported about the direct contribution of different soluble factors to ASC immunomodulatory activity, including, among others, hepatocyte growth factor (HGF) (Puissant et al. 2005; Cui et al. 2007; DelaRosa et al. 2009), IL-10 (Puissant et al. 2005; DelaRosa et al. 2009; Ji et al. 2015; Jang et al. 2020), human leukocyte antigen G 5 (HLA-G5) (Menta et al. 2014; Ceccarelli et al. 2020; Zoehler et al. 2022), and TGF-β1 (Puissant et al. 2005; Cui et al. 2007; DelaRosa et al. 2009; Serena et al. 2016; Jang et al. 2020).

One of the most relevant mechanisms involved in hASCs immunosuppression is indoleamine 2, 3-Dioxygenase 1 (IDO), an enzyme that degrades tryptophan to kynurenine (Yoo et al. 2009; DelaRosa et al. 2009; Menard et al. 2013; Menta et al. 2014; Rubtsov et al. 2017; Kim et al. 2018; Torres Crigna et al. 2020; Fiori et al. 2021; Mckinnirey et al. 2021). IDO is not constitutively expressed in MSC, but it is induced by IFN-ɤ (DelaRosa et al. 2009; Torres Crigna et al. 2020; Mckinnirey et al. 2021). To confirm the role of IDO in mediating centrally hASCs immunosuppression, PBMC proliferation assays were carried out in the presence or absence of 1-MT, an inhibitor of IDO activity. The addition of 1-MT restored PBMC proliferation. Additionally, clones of hASCs overexpressing IDO were able to potentially inhibit PBMC proliferation (DelaRosa et al. 2009). In another report, higher IDO mRNA and protein levels were detected in contactless- than contact-dependent hASC immunosuppression (Rubtsov et al. 2017).

Menta et al. (2014) investigated the relevant roles of tryptophan (Trp) depletion and/or kynurenine accumulation on the inhibition of T cells by hASCs. It was found that Trp supplementation in the medium of T cells co-cultured with hASCs impaired the capacity of the latter to reduce T cell proliferation and viability. The addition of kynurenine variably inhibited T lymphocyte proliferation, suggesting that the sensitivity of lymphocytes to kynurenine may be donor-dependent. Such results confirmed that Trp deprivation rather than kyn accumulation is the main factor mediating the immunosuppressive capacity of ASCs. Noteworthy, adding Trp in the proliferation assays reversed the hASC antiproliferative effect despite the presence of anti-inflammatory mediators in the medium as soluble HLA-G 1/5 and PGE2 (Menta et al. 2014). Direct and transwell co-culture with hASCs significantly inhibited the proliferation of mitogen-stimulated human, not murine, lymphocytes via induced IDO expression, tryptophan depletion, and increased kynurenine secretion, however, nitrite secretion was not involved in hASC immunosuppression (Torres Crigna et al. 2020). Such findings demonstrate that Trp metabolism/catabolism is the major mechanism mediating the antiproliferative effect of hASCs on T lymphocytes, supporting the presence of hierarchy among different immunomodulators used by hASCs (Menta et al. 2014).

Other investigators explored the role of some surface molecules in the hASCs’ immunomodulatory effects (Shi et al. 2011; Jang et al. 2014; Xishan et al. 2015; Rubtsov et al. 2017; Zhou et al. 2018). hASCs facilitated the immunosuppressive effect of cyclosporine A on T lymphocytes via their expression of a high level of Jagged-1. Jagged-1 induced Notch signaling in T lymphocytes, reducing NF-κB nuclear translocation and activity, and that was associated with decreased transcription and production of IL-2 and IFN-ɤ (Shi et al. 2011). In another study, it has been reported that hASCs exerted immunosuppressive effects on PHA-activated T cells via a Jagged 2-dependent (Notch signaling ligand 2) manner (Xishan et al. 2015). The stable expression of human leukocyte antigen G 1 (HLA-G1) enhanced the hASCs’ antiproliferative effect on PHA-stimulated PBMCs (Yang et al. 2012). Recently, it has been reported that the membrane-bound form of HLA-G1 was constitutively expressed by MSCs from different sources, including AT, and the addition of IFN-ɤ did not influence its expression (Zoehler et al. 2022).

Programmed death -ligand 1 (PD-L1/B7-H1), a negative regulatory molecule, is known to regulate T cell activation and tolerance via programmed death 1 (PD-1) (Sunshine and Taube 2015). PD-L1 is lowly expressed by naive hASCs; however, it is robustly induced by the incubation of ASCs with IFN-ɤ (Kronsteiner et al. 2011; Menard et al. 2013; Jang et al. 2014). Blocking experiments proved that the PD-L1 pathway mediated the antiproliferative effect of various MSC populations, including hASCs, on PHA-activated T cells, in a direct cell-to-cell contact setting; however, this effect was determined by the MSC expression proportion of HLA-DR (Jang et al. 2014). Zhou et al. (2018) investigated the immunomodulatory effects of hASCs on T-cell subsets and the related underlying molecular mechanisms. hASCs exerted immunosuppressive effects on CD4 and CD8 T cells via, at least partially, the reduction of NF-κB activation mediated by PD-L1/PD-1 and galactin 9 (Gal-9)/T-cell immunoglobulin and mucin-containing protein 3/(TIM-3) pathways with more predominant effect for the former axis (Zhou et al. 2018). CD200 on MSCs binds to CD200R on immature DCs, inhibiting their maturation into fully differentiated DCs with high surface expression of CD83 and CD86 (Zhao et al. 2021).

MSCs express chemokines and adhesion molecules that mediate their potential to establish cell membrane interactions with activated lymphocytes to abrogate their functions (Majumdar et al. 2003; Ren et al. 2008). One of the highly suggested adhesion molecules underlying contact-dependent hASC immunosuppression is the intracellular adhesion molecule-1 (ICAM-1)/ (CD54). CD200^low^ CD274^low^ ASCs expanded in clinical grade platelet lysate (PL) strongly inhibited the proliferation of activated CD3 T cells, and CD54 was suggested to be implicated as a dominant factor in this effect (Menard et al. 2013). In cope, the expression of ICAM-1 increased significantly by hASCs after co-culture with PBMCs (either stimulated or non-stimulated) (Rubtsov et al. 2017). hASCs were found to downregulate ICAM-1 expression in activated T cells, in correlation with the downmodulation of the T-cell activation marker CD25; however, they upregulate ICAM-1 expression in the resting T cells. ICAM-1 blocking restored CD25 expression on activated lymphocytes abrogating the immunosuppressive effect of hASCs. Noteworthy, ICAM-1 blocked reversed the hASCs’ stimulatory effect on the resting CD4 T cells (Rubtsov et al. 2017).

Taken together, such data provide insights into mechanisms of hASC-mediated immunosuppression via either release of soluble mediators, IDO, PGE2, IL-6, and LIF are among the leading ones. In addition to the induction of extracellular vesicles (exosomes) (Dabrowska et al. 2021) and the surface expression of immunosuppressive molecules including, among others, CD54, CD274, and galectins. Further studies, to insight into the molecular mechanisms and signaling pathways that are activated in both hASCs and diverse immune cells in inflammation, would direct and potentiate hASC immunomodulatory therapy.

## hASC induction of Tregs: specific immunomodulatory mechanism

One of the potential functions by which hASCs can modulate immune responses is their capability to induce and/or expand a regulatory and anti-inflammatory T cell phenotype (CD4^+^CD25^high^FoxP3^+^) (Najar et al. 2010b; Ivanova-Todorova et al. 2012; Engela et al. 2013; Karaöz et al. 2016; Skalska et al. 2017; Yousefi et al. 2019; Bi et al. 2020; Fakhimi et al. 2019; Fiori et al. 2021; Kuca-Warnawin et al. 2021a; Mahmoud et al. 2023). Regulatory T cells (Tregs) have been shown to restrain excessive inflammatory responses in autoimmune diseases. CD4^+^CD25^high^FoxP3^+^ T cells, as one of CD4^+^ FOXP3^+^ Tregs subpopulations, can originate directly from the thymus and are designated as natural (nTregs) or can differentiate under the influence of cytokines in the periphery and are called induced (iTregs) (Ziegler and Buckner 2009). Cell–cell contact, PGE2, TGF-β1 (English et al. 2009), IL-6 (Crop et al. 2010; Ivanova-Todorova et al. 2012; Sivanathan et al. 2015), PD-L1 involvement (Amarnath et al. 2011), Notch signaling (Rashedi et al. 2017), LIF (Najar et al. 2010b), and/or IDO (Ge et al. 2010), are among the crucial mechanisms underlying MSC-mediated induction of Tregs. Reprogramming of antigen-presenting cells such DCs (Zhao et al. 2012), or macrophages (Melief et al. 2013b) toward tolerogenic phenotypes has been evidenced as a potential mechanism by which MSCs from sources, rather than AT, generate Tregs. It is commonly accepted that the CD4^+^FoxP3^+^ T cells exert their immunosuppressive effects through the secretion of TGF-β1 and/or IL-10 (Jiang and Chess 2004; English et al. 2009).

Najar and his colleagues (2010b) reported that hASCs and WJ-MSCs, not BM-MSCs, maintained and promoted the expansion of CD4^+^ CD25^+^ FOXP3^+^ Tregs upon co-culture with PHA/IL-2 activated T cells independently of the MSC/T-cell ratio, with a better effect by hASCs (Najar et al. 2010b). On the contrary, the percentage of Tregs exhibiting the phenotype CD4^+^ CD25 FOXP3^+^ was greatly elevated with the strongest effect in co-cultures of PHA-CD3^+^ T cells with BM-MSCs, then WJ-MSCs and finally hASCs with the weakest immunosuppressive activity (Karaöz et al. 2016).

In another report, hASCs enabled a specific proliferation of activated CD4^+^CD25^+^ T cell fraction and affected their composition. hASCs induced the expression of CD127 (IL-7R) expanding the CD4^+^CD25^+^CD127^+^FoxP3^−^ (probably memory T cells) at the expense of CD4^+^CD25^+^CD127^−^FOXP3^−^ (effector T cells) via mostly IL-7 dependent mechanism (Fiori et al. 2021). Importantly, upon CD3 stimulation, the former subset secreted effector cytokines of the T helper 2 (Th2) cells mainly, suggesting their function in controlling inflammatory responses (Narsale et al. 2018). The modulation of effector/memory T cell subsets represents a novel point of interest in the study of hASCs immunomodulation (Fiori et al. 2021).

Kuca-Warnawin et al. reported that hASCs were not able to promote Tregs formation in the absence of accessory mononuclear cells as monocytes. Therefore, in co-culture with stimulated PBMCs, not enriched CD4 T cells, hASCs significantly increased the frequency of CD4^+^ CD25^high^ FOXP3^+^ T cells. Importantly, the presence of inflammatory disease as ankylosing spondylitis did not affect such property (Kuca-Warnawin et al. 2021a). In a partial contradiction with that, ASCs isolated from patients with obesity and T2D or nondiabetic donors with normal weight, were able to comparably increase the frequency of CD4^+^ CD25^+^ FOXP3^+^ even in coculture with anti-CD3/CD28- purified CD4 T cells (Mahmoud et al. 2023).

nTregs and ASC-induced Tregs are distinct from each other in their place of origin, the stability of FOXP3 expression, and the methylation pattern of the Tregs-specific demethylated region (TSDR) in the *FOXP3* gene. nTregs develop intra-thymically, constitutively express FOXP3 and have a fully demethylated FOXP3 TSDR. In contrast, iTregs development takes place in the periphery, their FOXP3 expression is inducible, and their FOXP3 TSDR is fully methylated (Baron et al. 2007; Bilate and Lafaille 2012). Engela et al. (2013) investigated whether perirenal hASCs can generate functional de novo iTregs from allo-stimulated CD25^−/dim^ effector T cells and to find evidence for the mechanisms involved in ASC-mediated Tregs induction. hASCs suppressed the proliferation of allo-stimulated CD25^−/dim^ effector cells and induced a 2.1-fold increase in the percentage of CD25^+^CD127^−^FOXP3^+^ cells within the CD4 T cells via IL-2 dependent pathway (Engela et al. 2013). The hASC-iTregs inhibited effector cell proliferation as effectively as nTregs. The vast majority of cells within the iTregs fraction had a methylated FOXP3 gene TSDR indicating that they were not of nTregs origin (Engela et al. 2013).

Some authors reported that hASCs promote the expansion of CD4^+^ CD25^+^ T cells and increase the frequency of Tregs in non-stimulated co-culture settings (Crop et al. 2010; Ivanova-Todorova et al. 2012; Frazier et al. 2014; Fiori et al. 2021). hASCs had the potential to activate and induce proliferation of resting allogeneic CD4 T cell fraction of PBMCs (Crop et al. 2010), or naïve CD4 T cells, derived from breast cancer patients (Frazier et al. 2014) in direct and/or transwell co-culture. The activated population exhibited Tregs phenotype and immunosuppressive activities. Conditioned medium (CM) of hASCs increased the frequency of Tregs producing IL-10 (Ivanova-Todorova et al. 2012). hASCs induced significant proliferation and induction of Tregs (CD4^+^CD25^+^CD127^low^FOXP3^+^) specifically in direct co-cultures, supported by a high concentration of TGF-β1 (Fiori et al. 2021).

The dual effect of hASCs of inhibiting immune cell proliferation while generating de novo immunosuppressive cells emphasizes their potential as a cellular immunotherapeutic agent (Engela et al. 2013). However, the potential of hASCs to induce Tregs in preclinical disease models and clinical studies is still poor and it needs further investigation. The synergistic immunoregulatory effects for a combinatorial approach of hASCs and hASC-induced Tregs in autoimmune and/or inflammatory diseases might provide a promising immunotherapeutic modality (Negi and Griffin 2020), and it represents an interesting research area.

## hASCs implication for T2D treatment

Preclinical studies (Table 1) have shown promising results for the therapeutic effect of hASCs in T2D animal models, depending at least partially on their anti-inflammatory properties (Ji et al. 2015; Shree and Bhonde 2016; Lee et al. 2017; Domingues et al. 2019; Jaber et al. 2021). hASCs isolated from human orbital fat tissues were able to correct the inflammatory and metabolic imbalances in HFD- or obesity-induced diabetes (Ji et al. 2015). In that study, systemic transplantation of hASCs reduced inflammatory cell infiltration in AT of diabetic mice and serum levels of adipokines, including leptin and TNF-ɑ. hASCs supported pancreatic islet growth by direct differentiation into insulin-producing cells and by mitigating the cytotoxicity of IL-1 and TNF-ɑ in the pancreas. Human IDO and IL-10 were upregulated in the treated mice pancreatic tissues pointing out their possible role in the observed hASCs anti-inflammatory and reparative effect. Additionally, hASCs improved glucose tolerance as they upregulated glucose transporter 4 (GLUT4)-mediated glucose uptake in the diabetic skeletal muscles via expression of IL-1 receptor antagonist (IL-1RA) (Ji et al. 2015).

**Table 1:** Preclinical studies of hASC-based therapy in T2D animal models

**Reference**	**Disease model**	**Sample size (experimental vs control)**	**Native or manipulated hASCs**	**Regimen**	**Route of administration**	**Follow-up duration**	**The observed immunoregulatory therapeutic effect**
Ji et al. (2015)	T2D HFD-induced diabetes)/mice for continuous 12 weeks	*n* = 10/group	native hASCs	Two episodes of 4.2 × 10^7^ cells/kg body weight in .2 ml PBS with an interval of eight weeks	IV	12 weeks	hASCs ameliorated pancreatic tissue inflammation and insulin intolerance and glucose uptake in insulin-sensitive tissues (liver and skeletal muscle) possibly via release of IL-1Ra and expression of immunomodulators such as IDO, IL-10, and sTNF RII
Shree and Bhonde (2016)	T2D HFD-induced diabetes)/mice for continuous 10 weeks	*n* = 4/group	hASCs were naïve or preconditioned with metformin	5 × 10^5^ cells/animal	IM	ND	Administration of hASCs reduced systemic inflammation (serum IL-6 level) and expression of inflammation genes (*IL-6* and *PAI-1*) in the liver. They also upregulated glucose uptake and GLUT4 expression in skeletal muscles
Lee et al. (2017)	HFD-induced obesity and metabolic syndrome	*n* = 6	Naive hASCs	1 × 10^6^ cells/kg/ weight for 10 weeks with a 2-week interval	IP	ND	ASCs modulated glucose intolerance and inflammation in HFD-induced obesity and metabolic syndrome model
Domingues et al. (2019)	T2D (HFD-induced DM/mice)	*n* = 3 for CAT-ASCs or 4 for SOD2-ASCs group	SOD2 or CAT-overexpressing hASCs	1.5 million modified hASCs in .1 or .2 ml PBS/animal	IP	4 weeks	Genetically modified hASCs ameliorated glucose imbalance and systemic inflammation (reduced plasma TNF-ɑ level). They also ameliorated fatty liver ad hyperplasia of fat cells in omental fat
Jaber et al. (2021)	T2D HFD-induced obese and diabetic mice)	*n* = 6/group	Naive hASCs	4.2 × 10^7^ cells twice with 10 weeks-interval	IP	6 weeks	ASCs improved glucose tolerance in obesity-induced diabetic mice. In addition, ASCs caused significant attenuation in the levels of inflammatory mediators, TNF-ɑ and IL-6, in HFD-fed mice

*AT* adipose tissue, *CAT* catalase, *GlUT 4* glucose transporter 4, *hASCs* human adipose tissue-derived mesenchymal stromal/stem cells, *HFD* high-fat diet, *IDO* indoleamine 2,3 dioxygenase 1, *IM* intramuscular, *IP* intraperitoneal, *IV* intravenous, *IL-1Ra* interleukin 1 receptor antagonist, *IL-6* interleukin 6, *ND* not defined, *PAI-1* plasminogen activator inhibitor 1, *sTNF RII* soluble tumor necrosis factor receptor, *SOD2* superoxide dismutase 2, *TNF-ɑ* tumor necrosis factor-alpha

In another study, intramuscular transplantation of hASCs in HFD-diabetic mice led to a reversal of hyperglycemia, hyperinsulinemia, and triglyceridemia. That was associated with reduced serum IL-6 level, upregulated GLUT4 expression in the skeletal muscles, and downregulated expression of genes involved in inflammation in the liver as IL-6 and plasminogen activator inhibitor-1 (*PAI-1*). Importantly, metformin preconditioned ASCs exhibited potentiated anti-diabetic and anti-obesity effects (Shree and Bhonde 2016). Intraperitoneal (IP) administration of hASCs effectively treated experimental obesity-associated diabetes via correcting glucose intolerance, reducing the level of diabetogenic pro-inflammatory cytokines IL-6 and TNF-ɑ (Jaber et al. 2021), decreasing body weight, and modulating the expression of the adipokines adiponectin and leptin (Lee et al. 2017).

The anti-diabetic effect in experimental T2D models was not restricted to hASCs, but it extended to comprise their cell-free derivatives. CM of hASCs restored insulin signaling and glucose uptake in an insulin-resistant cell model. Such effects were mediated via upregulated expression of GLUT4 and phosphorylated AKT proteins with a concomitant decrease in inflammation markers IL-6 and PAI1 (Shree and Bhonde 2016).

Few clinical trials have shown improved metabolic indices such as decreased glycated hemoglobin and improved C peptide levels (Dang et al. 2017; Qi et al. 2019), and many questions remain to be addressed before the establishment of routine hASCs treatments for T2D. Patient-derived (autologous) rather than allogeneic MSC may be a relatively safer choice in clinical perspectives, to avoid anti-donor immune responses in some cases (Rossignol et al. 2009; Griffin et al. 2013). An interesting study has evaluated the efficacy and safety of autologous BM-MSCs transplantation in T2D patients to conclude that the clinical outcomes are greatly affected by patient characteristics involving disease duration and BMI (Nguyen et al. 2021). The autologous BM‐MSCs transplantation in T2D patients was efficient in recipients with a disease duration of ˂10 years and BMI < 23. T2D abrogated the glycolysis, mitochondria respiration of BM‐MSCs, and ATP generation and induced the accumulation of mitochondria DNA mutation; thus, T2D-associated altered cell metabolism may underlie attenuated patient MSC functions (Nguyen et al. 2021).

## Impact of obesity and T2D on hASC immunoregulatory properties

The biological and functional characteristics of MSCs, isolated from patients with diabetes mellitus, comprising phenotype, proliferation, survival, differentiation, angiogenic potential, and fibrinolytic activity, have been studied, documenting controversial data (Mahmoud et al. 2019). A number of studies explored the impact of obesity (Table 2) (Eljaafari et al. 2015; Strong et al. 2016; Serena et al. 2016; Usha Shalini et al. 2017; Harrison et al. 2020; Zhu et al. 2021; Juntunen et al. 2021), T2D (Mancini et al. 2017; Aliakbari et al. 2019; Wang et al. 2020; Abu-Shahba et al. 2021; O’Donnell et al. 2022), or both (Serena et al. 2016; Mahmoud et al. 2023) (Table 3) on the immunomodulatory properties of hASCs. Inconsistent results, from being intact (Usha Shalini et al. 2017; Wang et al. 2020; Juntunen et al. 2021) to slightly affected (Mahmoud et al. 2023), or severely altered (Eljaafari et al. 2015; Strong et al. 2016; Serena et al. 2016; Mancini et al. 2017; Aliakbari et al. 2019; Harrison et al. 2020; Abu-Shahba et al. 2021; Zhu et al. 2021) immunoregulatory properties of hASCs from patients with obesity and/or T2D, have been reported, and are illustrated in more detail below.

**Table 2:** Studies investigating the immunomodulatory properties of hASCs isolated from donors with obesity

**Ref**	**AT site location**	**Age (range or mean ± SEM)**	**Metabolic Indices (values in range or mean ± SEM)**	**BMI (range or mean ± SEM)**	**No. of obese subjects**	**The studied immune-related properties and/or functions**	**The relevant immunomodulatory findings**
Eljaafari et al. (2015)	Visceral and subcutaneous AT	ND	ND	ND	ND	T cells differentiation	Obese not lean ASCs promoted Th17 polarization in IL-1β-dependent, not IL-6, manner; however, they reduced Th1 cytokines. Increased IL-17A production inhibited adipogenesis and promoted insulin resistance in adipocytes
Serena et al. (2016)	Subcutaneous and visceral abdominal AT	39 ± 8.9	-HOMA-IR 2.84 ± 0.2	33.1 ± 2.1	*n* = 4	-Basal expression of pro-inflammatory cytokines as IL-1β, TNF-ɑ, MCP-1 and critical inflammasome components and anti-inflammatory cytokines as TGF-β1 and IL-10 by obese vs lean ASCs -The potential of CM of obese vs lean ASCs to suppress the proliferation of PBMCs in MLR	Obese ASCs exhibited impaired antiproliferative effect due to elevated expression of IL-1β, inflammasome activation, however, reduced expression of TGF-β1 by dASCs
Strong et al. (2016)	Subcutaneous Abdominal AT	42.5 ± 8.9	ND	32.7 ± 3.7	*n* = 6	-Potential to suppress murine T cell proliferation The therapeutic efficacy in EAE	-Obese ASCs failed to halt the disease progression in CNS of EAE -Obese ASCs enhanced proliferation and differentiation of CD4 and CD8 T cells
Usha Shalini et al. (2017)	Subcutaneous Abdominal AT	18–62	ND	Morbidly obese, however, exact BMI is ND	*n* = 10	- Potential to suppress CD3 T cells proliferation Isolated from RA patient -induction of Tregs -Modulation of cytokines production	Morbidly obese ASCs were able to inhibit proliferation of CD3 T cells, derived from RA patients, in a dose-dependent fashion, however, they promoted that of CD4^+^ FOXP3^+^ T cells. They also downregulated the production of IFN-ɤ and TNF-ɑ, however, upregulated IL-10 by RA-CD3 T cells
Harrison et al. (2020)	Subcutaneous, abdominal AT	40.50 ± 7.46	ND	33.97 ± 3.11	*n* = 6 (pooled)	-The potential to modulate macrophage and microglia polarization toward anti-inflammatory phenotype -Modulation of gene expression of pro- and anti-inflammatory markers by macrophages -Modulation of Nitric oxide activity and phagocytic activity of macrophages	Obese human ASCs induced polarization of murine microphages and microglia toward a pro-inflammatory phenotype
Juntunen et al. (2021)	Subcutaneous AT	33.5–34.1	- HOMA-IR range 0.6–2.7 -Plasma adiponectin: 1310–4930	25.2–44.2	*n* = 5	-Immunogenic effect -Suppression of PBMC proliferation in MLR	Leaner and heavier WD donors were hypoimmunogenic, however, heavier WD donors showed superior immunosuppressive capacity
Zhu et al. (2021)	ND	57.0 ± 2.6	50% of obese subjects had hypertension, 66% had T2D, and 66% suffered from hyperlipidemia	42.9 ± 1.1	*n* = 6	Potential to modulate the polarization of macrophage in inflammation toward anti-inflammatory phenotype (M2) at the expense of inflammatory phenotype M1	ASCs isolated from obese donors had blunted immunomodulatory potential on activated macrophages TNF-α levels were four-fold higher in CM collected from obese than from lean ASCs

*AT* adipose tissue, *BMI* body mass index, *HOMA-IR* homeostatic model assessment for insulin resistance, *IL-6* interleukin 6, *IL-1β* interleukin 1 beta, *IL-17A* interleukin 17A, *IL-10* interleukin 10, *MCP-1* monocyte chemotactic protein 1, *MLR* mixed lymphocyte reaction, *ND* not defined, *RA* rheumatoid arthritis, *SEM* standard error mean, *Treg* T cell with immune regulatory functions, *WD* weight- discordant, *TNF-ɑ* tumor necrosis factor-alpha, *TGF-β1* transforming growth factor beta 1

**Table 3:** Studies investigating the immunomodulatory properties of hASCs isolated from patients with T2D

**Ref**	**AT site location**	**Age (years) (range or mean ± SEM/SD)**	**Metabolic Indices (range or mean ± SEM)**	**BMI (range or mean ± SEM)**	**No. of T2D patients**	**The studied immune-related properties and/or functions**	**The relevant immunomodulatory findings**
Serena et al. (2016)	Subcutaneous and visceral abdominal AT	45 ± 10.3	-HOMA-IR 3.18 ± 0.5 - HbA1c 6.1–6.9	35.3 ± 1.5	*n* = 4	-Basal expression of pro-inflammatory cytokines as IL-1β, TNF-ɑ, MCP-1 and critical inflammasome components and anti-inflammatory cytokines as TGF-β1 and IL-10 by obese T2D vs lean healthy ASCs -The potential of CM of T2D obese vs healthy lean ASCs to suppress the proliferation of PBMCs in MLR -The effect of priming with TGF-β1 and IL-1RA on the immunomodulatory dysfunction of dASCS	T2D obese ASCs exhibited impaired antiproliferative effect on T cells and M2 promoting effect. The immunomodulatory dysfunction of dASCs was attributed to elevated inflammasome-mediated expression of IL-1β, however, reduced expression of TGF-β1 by dASCs Importantly, priming of dASCs with TGF-β1 and IL-1RA reversed their immunomodulatory dysfunction
Mancini et al. (2017)	Subcutaneous AT from pts undergoing elective cardiovascular	63.4 ± 13.3	ND	ND	*n* = 12	Potential of dASCs to suppress CD4 or CD8 T cells proliferation	Presence of T2D, in association with ATH, strongly reduced ASCs to suppress T cell proliferation relative to ATH alone or healthy groups
Aliakbari et al. (2019)	omental AT	40–55	FBG ≥ 126	ND	*n* = 7	- Suppression of PBMC proliferation in MLR - Gene expression levels of COX-2, IL-6, TNF-α and TGF-β1 in PBMC mono-cultures and co-cultures with ASCs - Protein levels of IFN-ɤ and IL-10 in co-cultures in PBMC mono-cultures and co-cultures with ASCs	T2D attenuated the potential of ASCs to suppress PBMC proliferation, IFN-ɤ, TNF-ɑ and IL-6 in MLR. The anti-inflammatory mediators TGF-β1 and COX-2 were less expressed in dASCs than ndASCs co-cultures
Wang et al. (2020)	peripancreatic AT	50.33 ± 1.33	- HbA1c 27.17 ± 0.48	27.17 ± 0.48	*n* = 3	-Suppression of PHA-activated CD3^+^ T cells	Comparable to ndASCs, dASCs exerted anti-proliferative effect on PHA-activated CD3^+^ T cells
Abu-Shahba et al. (2021)	Subcutaneous Abdominal AT	30–65	ND	≤ 33	*n* = 13	-Surface expression of immunomodulators as CD200, CD276, and CD274 -Gene expression of pro-inflammatory as TNF, IL-6, IL-1β, anti-inflammatory as IDO, PTGS2, IL-10 and TSG-6 and adipokines as adiponectin, visfatin and leptin -Response to IFN-ɤ licensing	- T2D was found to significantly suppress the expression of the surface immunomodulators, CD200 and CD276, and the soluble ones, IL-1RA and IDO by hASCs. However, T2D ASCs significantly expressed higher levels of the diabetogenic mediator, IL-1β gene and protein, than ndASCs -Both dASCs and ndASCs responded similarly to IFN-ɤ priming and upregulated significantly the expression of the immunomodulators IDO, IL-1RA, and CD274
O’Donnell et al. (2022)	IPFP	53.3 ± 11.3 (T2D group), 65.7 ± 4.50 (pre-T2D group)	ND	32.1 ± 3.6 (T2D group), 36.6 ± 6.0 (pre-T2D group)	*n* = 3 (T2D group), *n* = 3 (pre-T2D group)	-The expression profiles of inflammatory and adipokine-related genes in IPFP-ASCs of non-diabetic, Pre-T2D, and T2D donors at basal level and in response to IL-1β stimulation -The immunomodulatory functions of the three studied ASC groups on monocyte-derived macrophages (M0, M1, or M2)	-The study demonstrated that pre-T2D not T2D ASCs, compared to non-diabetic, had significantly increased the gene expression of COX-2 and FOXG1 and the secretion of PGE2 in response to IL-1β stimulation -Interestingly, M1 macrophages exhibited a significant reduction in expression of the pro-inflammatory markers TNF-α and IL-6 when co-cultured with pre-T2D IPFP-ASCs -The aberrant expression of COX-2 and increased secretion of PGE2 in IPFP-ASCs could induce a cartilage catabolic state enhancing the risk of OA in pre-T2D patients
Mahmoud et al. (2023)	Subcutaneous Abdominal AT	50–62	ND	32.6–50.9	*n* = 9	-The expression of the immunomodulators IDO, CD274, and CD54, among other immune-related markers, by dASCs and ndASCs in inflammation -The effects of dASCs vs ndASCs to modulate anti-CD3/CD28 proliferation and activation -The potential of dASCs and ndASCs to increase Treg frequency and suppress the intracellular IFN-ɤ production by CD4 T cells -The immunomodulatory effects of dASCs and ndASCs on the cytokines secretion pattern in coculture with anti-CD3/CD28-activated CD4 T cells	-The study demonstrated that the expression of CD54, CD274, and IDO, in inflammation, was significantly upregulated in ASCs, with no significant differences between ndASCs and dASCs -dASCs retained the potential to significantly suppress CD4 T-cell proliferation, with a slightly weaker inhibitory effect than ndASCs, which was associated with significantly reduced abilities to decrease IL-2 production and increase IL-8 levels in cocultures. Such attenuated potentials were significantly correlated with increasing body mass index - dASCs and ndASCs comparably reduced CD4 T cell viability, HLA-DR expression, and interferon-gamma production and conversely increased CD69 expression, IL-17A production, and the Tregs frequency -Maintained dASC immunosuppressive functions were attributed at least partially to the nonsignificant difference in the basal expression level of TGF-β1 between the dASCs and ndASCs

*AT* adipose tissue, *ATH* atherosclerosis, *BMI* body mass index, *CD* cluster of differentiation, *COX-2* cyclooxygenase 2, *dASCs* ASCs from patients with obesity and T2D, *FBG* fasting blood glucose, *FOXG1* Forkhead box G1, *HOMA-IR* homeostatic model assessment for insulin resistance, *HbA1c* glycated hemoglobin, *HLA* human leukocyte antigen, *IDO* indoleamine 2,3 dioxygenase, *IFN-ɤ* interferon-gamma, *IL-10* interleukin 10, *IL-6* interleukin 6, *IL-1β* interleukin 1 beta, *IL-1RA* interleukin 1 receptor antagonist, *IL-2* interleukin 2, *IL-8* interleukin 8, *IL-17A* interleukin 17A, *IPFP* infrapatellar fat pad, *MCP-1* monocyte chemotactic protein 1, *MLR* Mixed lymphocyte reaction, *ND* not defined, *ndASCs* ASCs from nondiabetic donors, *OA* osteoarthritis, *PGE-2* prostaglandin E2, *PHA* phytohemagglutinin, *Pre-T2D* pre-diabetic, *SEM* standard error mean, *SD* standard deviation, *T2D* type 2 diabetes, *TNF-ɑ* tumor necrosis factor alpha, *TGF-β1* transforming growth factor beta 1, *TSG-6* tumor necrosis factor-stimulated gene 6

Obesity has been reported to deteriorate the immunosuppressive capacity of hASCs (Strong et al. 2016; Serena et al. 2016). Obese, not lean, hASCs promoted IR in adipocytes. Co-culture of lean or obese hASCs with anti-CD3/CD28 activated PBMCs revealed that obese not lean hASCs induced Th17 promotion and monocyte activation. Cell-to-cell contact was needed to induce IL-1β secretion by PBMCs, which in turn induced IL-17A inflammation, despite reduced secretion of Th1 cytokines (IFN-ɤ and TNF-ɑ). Such a pro-inflammatory environment was thought to promote IR (Eljaafari et al. 2015). Lean vs obese hASC therapeutic efficacy in experimental autoimmune encephalomyelitis (EAE) was tested. Lean hASCs succeeded in relieving disease progression and symptoms, depending on their anti-inflammatory effects, in different stages of disease onset; however, obese counterparts failed to do, even though they enhanced inflammatory cell infiltration and demyelination of the central nervous system (CNS) and disrupted the lymphoid organs in EAE model. Additionally, CM collected from the obese ASCs markedly enhanced the proliferation and CD4 and CD8 differentiation of murine CD3^+^ T cells; whereas, CM from lean hASCs did not. Defective immunoregulation of obese ASCs was attributed to their pro-inflammatory conditions, and importantly IFN-ɤ stimulation of obese ASCs induced them to express higher levels of pro-inflammatory mediators, including among others IL-1, IL-6, IL-12, TNF-ɑ, and IL-8, and potentiated further reduction in their therapeutic effect in EAE model. The authors concluded that obese hASCs are a suboptimal therapeutic option for autoimmune diseases (Strong et al. 2016). Additionally, AT-MCs from obese donors promoted a pro-inflammatory phenotype in murine macrophage and microglial cells (brain-resident macrophages), as indicated by the upregulated expression of the pro-inflammatory genes, TNF-α, and increased nitric oxide pathway activity, and impaired phagocytosis and migration (Harrison et al. 2020). Another study confirmed the blunted immunomodulatory functions of ASCs from obese donors on macrophages by reducing the expression of arginase 1(M2 macrophage marker) and IL-1RA; however, increasing the release of TNF-α by macrophages activated with LPS and IFN-ɤ (Zhu et al. 2021).

On the contrary, obesity does not affect the immunosuppressive capacity of hASCs (Usha Shalini et al. 2017; Juntunen et al. 2021). The immunosuppressive capacity of lean vs. obese hASCs derived from weight-discordant monozygotic twin pairs has been recently compared. Obese hASCs exhibited a slightly higher, but significant, antiproliferative effect in a two-way, mixed lymphocyte reaction (MLR) (Juntunen et al. 2021). In another report, hASCs, collected from morbidly obese donors, have been reported to keep intact immunomodulatory properties. Morbidly obese hASCs inhibited the proliferation of CD3 T cells, derived from rheumatoid arthritis (RA) patients, in a dose-dependent fashion, however, they promoted that of CD4^+^ FOXP3^+^ T cells**.** They also downregulated the production of IFN-ɤ and TNF-ɑ, however, upregulated IL-10 by RA-CD3 T cells. Conclusively, morbidly obsess ASCs were able to modulate allogeneic T-cell immune responses in RA (Usha Shalini et al. 2017).

T2D has been reported to blunt the immunosuppressive properties of hASCs (Serena et al. 2016; Mancini et al. 2017; Aliakbari et al. 2019; Abu-Shahba et al. 2021). Mancini et al. (2017) addressed the potential of ASCs from patients with T2D alone or with atherosclerosis (ATH) to suppress the proliferation of allogeneic-activated CD4 T cells. The compromised T-cell antiproliferative effect of ASCs observed in T2D was more profound in the presence of ATH (Mancini et al. 2017). Serena et al. (2016) demonstrated that hASCs, isolated from subjects with obesity and T2D (dASCs), acquired a pro-inflammatory phenotype. It was characterized by elevated expression of the pro-inflammatory mediators TNF-ɑ, IL-1β, MCP-1, and IL-6 and was associated with activation of the inflammasome/IL-1β pathway. Importantly, CM from dASCs, exhibited a blunted immunosuppressive ability to inhibit lymphocyte proliferation in MLR or to promote the anti-inflammatory M2 macrophage phenotype. The significantly reduced expression of the anti-inflammatory cytokine TGF-β1, conversely, upregulated expression of IL-1β, were attributed to the defective immunosuppressive capability of dASCs. Noteworthy, the inflammatory phenotype of dASCs was associated with enhanced glycolysis and ATP generation (Serena et al. 2016). Later, such results were confirmed, and the potential of hASCs isolated from patients with T2D to suppress PBMC proliferation in MLR was significantly decreased (Aliakbari et al. 2019). Compared to nondiabetic ASC/PBMC co-cultures, T2D ASC/PBMC counterparts displayed Lower expression of the anti-inflammatory TGF-β1 and COX-2 and higher expression of the pro-inflammatory (IL-6, TNF-ɑ, and IFN-ɤ), mediators, indicating an impairment of hASCs from T2D patients in the modulation of inflammation (Aliakbari et al. 2019). Recently, it has been demonstrated that ASCs, derived from T2D subjects with BMI ≤ 33, exhibit a pro-inflammatory phenotype characterized by altered gene and secretory levels of pro-and anti-inflammatory mediators. T2D was found to significantly suppress the expression of the surface immunomodulators, CD200 and CD276, and the soluble ones, IL-1RA and IDO by hASCs. However, ASCs from T2D patients significantly expressed higher levels of the diabetogenic mediator, IL-1β gene, and protein, than ASCs from nondiabetic donors (ndASCs) (Abu-Shahba et al. 2021).

On the contrary, ASCs from patients with T2D alone (Wang et al. 2020) or with obesity (Mahmoud et al. 2023) preserved their immunosuppressive functions in vitro. ndASCs and dASCs exhibited comparable antiproliferative effects on PHA-activated CD3^+^ T cells. dASCs were isolated from T2D patients with a mean glycated hemoglobin (HbA1c) level ± SEM (7.03 ± 0.66) (Wang et al. 2020). Recently, it has been demonstrated that dASCs, from patients with obesity and T2D, retained the potential to significantly suppress CD4 T-cell proliferation, with a slightly weaker inhibitory effect than ndASCs, which was associated with significantly reduced abilities to decrease IL-2 production and increase IL-8 levels in cocultures. Such attenuated potentials were significantly correlated with increasing body mass index. dASCs and ndASCs comparably reduced CD4 T cell viability, HLA-DR expression, and interferon-gamma production and conversely increased CD69 expression, IL-17A production, and the Tregs frequency. In inflammation, the expression of CD54, CD274, and IDO was significantly upregulated in ASCs, with no significant differences between ndASCs and dASCs. These findings suggest that ASCs obtained from donors with obesity and T2D are receptive to the inflammatory environment and able to modulate CD4 T cells accordingly. That was attributed at least partially to the nonsignificant difference in the basal expression level of TGF-β1 between the dASCs and ndASCs (Mahmoud et al. 2023).

O’Donnell et al. (2022) analyzed the expression profiles of inflammatory and adipokine-related genes in infrapatellar fat pad (IPFP)-ASCs of non-diabetic, pre-diabetic (pre-T2D), and T2D donors. The study demonstrated that pre-T2D, not T2D ASCs, compared to non-diabetic, had exhibited a substantial decrease in levels of mesenchymal markers CD90 and CD105, and in response to IL-1β stimulation, they significantly increased the gene expression of COX-2 and Forkhead box G1 (FOXG1) and the secretion of PGE2. Interestingly, M1 macrophages exhibited a significant reduction in expression of the pro-inflammatory markers TNF-α and IL-6 when co-cultured with pre-T2D IPFP-ASCs. The aberrant expression of COX-2 and increased secretion of PGE2 in IPFP-ASCs could induce a cartilage catabolic state enhancing the risk of osteoarthritis in pre-T2D patients. However, increased FOXG1 expression does imply that pre-T2D IPFP-ASCs are exhibiting a diabetes-related genotype (O’Donnell et al. 2022).

The lack of experimental and donor-related parameters standardization (Tables 2 and 3) may attribute to the inconsistent results of literature studies addressing the impact of obesity and/or T2D on the immunosuppressive functions of hASCs.

## Impact of obesity and T2D on hASC antioxidative properties

hASCs have been reported to manage oxidative stress efficiently and resist ROS-induced cell death (Park et al. 2011), in addition to the constitutive expression of antioxidant enzymes such as superoxide dismutases (SODs), catalase, and glutathione peroxidase (Abu-Shahba et al. 2020). Moreover, they have antioxidant and anti-apoptotic functions to protect mature cells from oxidative damage (Hou et al. 2022). CM from ASC cultures was found to mitigate oxidative stress and improve insulin sensitivity in an insulin-resistant cell model (Sanap et al. 2020).

Oxidative stress may result from the accumulation of ROS and/or impairment of antioxidant mechanisms, and it affects MSC characteristics and functions (Denu and Hematti 2016). ROS are molecules derived from oxygen (O2) that can readily oxidize other molecules. Most intracellular ROS, generated in mitochondria, is derived from superoxide anion, which is generated by the one-electron reduction of O2. Superoxide anion is converted to hydrogen peroxide (H2O2) by SODs (Sena and Chandel 2012).

In the context of T2D, it is expected that the mal environment of AT in individuals with obesity and T2D may increase the accumulation of intracellular ROS in hASCs. That may affect dASC stemness status, multipotency, secretory activity, and mitochondrial integrity (Kornicka et al. 2018). hASCs from subjects with T2D had impaired antioxidative protection resulting in mitochondrial dysfunction, senescent phenotype, and reduced sirtuin one gene (SIRT-1) expression. Strong evidence of the positive effect of decreased SIRT-1 activity on the development of IR and obesity, and then T2D (Alicka et al. 2019). In another report, higher levels of basal and stress-responsive ROS were generated by dASCs than by ndASCs, however, not significantly. Importantly, a significant positive correlation was observed between the population doubling time, as indices of growth kinetics, and basal ROS level (Mahmoud et al. 2023). Such findings show that the hyperglycemic milieu of T2D might affect the basal oxidative stress level of ASCs and so on their proliferation potential in vitro.

## Preconditioning strategies to improve dASCs immunomodulation in clinical settings

Strategies to improve the therapeutic efficacy of dASCs include preconditioning/priming strategies of cells with external stimuli, including a growth factor, a pro-inflammatory cytokine, or a chemical substance. The combined treatment with IL-1RA and TGF-β1 was able to reverse the dysfunctional immune behavior in dASCs (Serena et al. 2016).

Treatment of MSC by pro-inflammatory cytokines is one of the main approaches to boosting hASCs immunomodulation (Ceccarelli et al. 2020). It has been reported that priming of hASCs with IFN-ɤ (DelaRosa et al. 2009; Kronsteiner et al. 2011), TNF-ɑ (Domenis et al. 2018), IL-17 (Han et al. 2014; Pourgholaminejad et al. 2016), and/or IL-1β (Najar et al. 2019), induces hASCs to acquire an anti-inflammatory phenotype (Ceccarelli et al. 2020; Bernardo and Fibbe 2013). Priming with the master pro-inflammatory cytokine, IFN-ɤ, significantly augmented the anti-inflammatory hASC phenotype via induction of expression of various immunomodulators, importantly, IDO, COX-2 (DelaRosa et al. 2009; Kronsteiner et al. 2011)**,** CD274 (Jang et al. 2014), and CD54 (Rubtsov et al. 2017). Additionally, priming with a pro-inflammatory cytokine or hypoxia has been reported to reprogram MSC metabolism toward glycolysis, instead of oxidative phosphorylation and that provides an emerging avenue to enhance the immunomodulatory capacities of MSC (Li et al. 2012). dASCs and ndASCs exhibited a comparable potential to respond to inflammation. IFN-ɤ-primed ndASCs or dASCs upregulated the expression of the immunomodulators IDO, IL-1RA, and the surface protein CD274; however, downregulated IL-1β (Abu-Shahba et al. 2021). As well, they comparably upregulated the crucial ASC adhesion molecule CD54/ICAM-1 (Mahmoud et al. 2023).

Preconditioning hASCs with toll-like receptors (TLR) agonists is another priming approach (Lombardo et al. 2009). Baer et al. (2019) investigated the immunomodulatory potential of ASCs, isolated from kidney tumor patients, after stimulation with lipopolysaccharide (LPS) (TLR-4 ligand), lipoteichoic acid (LTA) (TLR-2 agonist), or a mixture of cytokines (IFN-ɤ, IL-1β, and TNF-α). LPS (from gram-negative bacteria) and cytokines, but not LTA (from gram-positive bacteria), induced a significant upregulation of mRNAs of ICAM-1, the pro-inflammatory cytokines IL-6 and TNF-α, and the chemokine MCP-1, in addition to IL-6 protein. Such results suggest the initiation of an inflammation-like response in LPS-treated hASCs (Baer et al. 2019). It has been reported that TLR3 or TLR4 activation enhances BM-MSC-mediated Tregs induction (Rashedi et al. 2017).

Treatment of hASCs with metformin enhanced their immunomodulatory functions in an animal model of lupus via the release of IDO, IL-10, and TGF-β1 by enhancing STAT1 expression, and that was dependent on the AMPK/mTOR pathway (Jang et al. 2020). All these studies recommend priming of hASCs with inflammatory or chemical molecules that could augment their immunosuppression and/or oxidative stress resistance in the settings of inflammatory diseases.

## Conclusion and future directions

Research indicates that ASCs possess immunoregulatory capacities on different immune cells, including lymphocytes, NK cells, DCs, and macrophages via direct and paracrine mechanisms. ASCs are a viable therapy for a wide range of inflammatory diseases such as T2D. Preclinical studies proved the potential of hASCs to ameliorate IR and attenuate inflammation in experimental T2D. For clinical application, autologous rather than allogenic ASCs may be a relatively safer choice from an immunological view. However, donors’ characteristics, such as BMI, health, and disease duration, are detrimental factors in ASCs therapy outcomes. Despite current investigations presenting a trend toward the altered immunoregulatory and antioxidative properties of ASCs, isolated from patients with T2D, they are still poor and lack standardization. Current data recommend that preconditioning of MSCs during the culture phase may alleviate the dysfunction of patient MSCs.

The lack of standardization may attribute to the inconsistent results of literature studies addressing the impact of obesity and/or T2D on the immunosuppressive functions of ASCs. Unified experimental conditions are required to validate the comparison of the reported results. Among those experimental conditions are ASC: immune cell ratio, ASC passage number, O2 tension, presence of accessory immune cells vs purified immune cell population, coculture medium, method of immune cell activation, and coculture time. Importantly, a study of patients with T2D should have matched age, sex, ethnicity, and BMI with nondiabetic donors. Full patients’ metabolic profiles including levels of glycated hemoglobin (HbA1c), fasting blood glucose, and c-peptide and IR indices, as well as patient lipid and biochemical profiles, should be included. Parameters such as disease duration and severity, the antidiabetic drugs regimen, donors’ inflammatory status, presence of other chronic conditions, and sample size need to be carefully considered in future studies focusing on exploring the impact of metabolic disturbances, specifically T2D, on the immunosuppressive functions of MSCs in vitro and in vivo.

The genetic and epigenetic landscape of ASCs, isolated from patients with T2D needs to be addressed. The metabolic activity and mitochondrial functions of ASCs derived from patients with T2D require intensive research. The immunomodulatory applicability of dASC-derived products, such as extracellular vesicles and exosomes, is poorly explored. It is, therefore, imperative to investigate the immunosuppressive functions of ASC-free derivatives in obesity and/or T2D. The impact of modulating the T2D patients’ body weight and lifestyle on improving the immunoregulatory capacities of dASCs can be addressed to open new avenues in autologous ASC therapy in T2D.

## Data Availability

Not applicable.
